# Priorities and Implementation Barriers in End-to-End Digital Transformation in the Pharmaceutical Industry: Sequential Mixed Methods Analysis

**DOI:** 10.2196/91531

**Published:** 2026-10-09

**Authors:** Isaac Rogelio Rodriguez-Chavez, Adama Ibrahim, Baris Ersezer, Angela Radcliffe, Victoria Gamerman, Bert Hartog, Lars Fogh Iversen, Marcus Schindler

**Affiliations:** 1 4Biosolutions Consulting, 510 Bradford Dr, Rockville, MD, 20850, United States, 1 2406725850; 2 Novo Nordisk, Måløv, Denmark; 3 Novartis, Basel, Switzerland; 4 How Mighty We Ventures, Huntingdon Valley, PA, United States; 5 RWD Insights, Stamford, CT, United States; 6 Hartog Digital Health Consulting, Middelburg, The Netherlands

**Keywords:** digital transformation, pharmaceutical industry, mixed methods research, implementation barriers, patient-centered care, clinical trials, Findable, Accessible, Interoperable, and Reusable, FAIR data principles, data governance, health equity, technology adoption, change management, organizational innovation

## Abstract

**Background:**

The pharmaceutical industry faces unprecedented challenges, including declining drug approval rates, plummeting research and development returns, and escalating protocol complexity. Digital transformation represents the most promising end-to-end approach for addressing these challenges, yet there is limited understanding of industry-wide priorities and implementation barriers.

**Objective:**

This study aimed to identify digital transformation priorities across diverse pharmaceutical industry stakeholders and to explore the specific barriers preventing successful implementation through a collaborative, mixed methods approach.

**Methods:**

The study used a sequential mixed methods design with three phases: (1) workshop 1 in Copenhagen, Denmark, with 47 participants randomly divided into 3 working groups, all discussing three main theme areas and generating 110 digital transformation topics; (2) a follow-up online survey of workshop 1 participants (administered 4 wk after workshop 1); and (3) workshop 2 in Boston, United States, with expert keynote and panel discussions exploring implementation challenges. Participants were recruited via convenience sampling through industry networks and professional associations. The study was conducted between March 2023 and June 2025 across international workshop sites in Copenhagen and Boston. Data sources included workshop outputs, survey responses, and transcribed panel discussions. Analysis involved thematic coding, convergence analysis, and tension identification.

**Results:**

Workshop 1 identified 19 common categories across all working groups, despite the 3 diverse starting themes. Workshop 1 produced 7 consolidated categories for theme 1 (desired outcomes), 5 for theme 2 (pathways), and 7 for theme 3 (measuring success), totaling 19 consolidated categories. The survey achieved a 55.3% (26/47 participants) response rate and identified patient-centered outcomes, FAIR (Findable, Accessible, Interoperable, and Reusable)–aligned data practices, collaboration, access and equity, and the reduction of patient burden as leading priorities. Workshop 2 identified 5 key implementation tensions: innovation speed vs safety standards, collaboration vs competition, technology vs change management, patient preferences vs operational efficiency, and data democratization vs privacy protection. Panel discussions provided deep insights into implementation challenges and solutions. The results obtained support exploratory generalization across comparable settings and do not establish causal effects.

**Conclusions:**

The pharmaceutical industry demonstrates strong consensus on patient-centric digital transformation priorities across the value chain, with universal recognition of the need for precompetitive collaboration. However, significant implementation barriers exist beyond technology, requiring integrated approaches to organizational change, regulatory innovation, and industry-wide standards development. Panel discussions and expert insights provide a pathway for continued collaboration and knowledge sharing. An incremental approach to sharing industry-wide knowledge is further recommended.

## Introduction

The global pharmaceutical industry confronts an intensifying economic sustainability crisis [1]. Success probabilities continue to decline, with fewer than 1 in 10 compounds entering clinical testing eventually approved, and oncology success rates as low as 1 in 20 [2]. Protocol complexity escalates relentlessly: data elements per protocol expanded from roughly 1 million to 3.6 million between 2010 and 2023 [3]. Amendment burdens, operational delays, and site enrollment failures that affect 40% of clinical trial sites compound these challenges [4]. The COVID-19 pandemic accelerated these trends, contributing to disruptions in clinical research operations [5-7]. Patients bear the ultimate cost through slower access to innovative therapies, uneven trial inclusion opportunities, and an increasing participation burden. This convergence of pressures demands a fundamental redesign rather than incremental optimization of legacy development models.

End-to-end approaches enhanced by digital transformation have emerged as the primary strategic lever for restoring pharmaceutical research and development productivity, resilience, and patient relevance. This coordinated integration of digital capabilities reshapes value creation, decision flow, and operating structures [5,8,9]. Digital health technologies in medical product development and modern trials, such as decentralized clinical trial (DCT) approaches, modern data platforms, AI and machine learning, advanced analytics, and automation, offer pathways to streamline evidence generation, broaden inclusion, and reduce operational friction [6,7,10-12].

The COVID-19 pandemic accelerated the adoption of DCTs enabled by digital health technologies, demonstrating feasibility for a broader research transformation [13]. Emerging economic evaluations also suggest that, in specific contexts, digital end points can be financially viable for sponsors, with modeling analyses indicating positive returns on investment when such measures are incorporated into phase 2 to 3 development [14].

Recent management science scholarship highlights that digital transformation in the pharmaceutical industry remains theoretically underdeveloped, with most research focusing on technology capabilities rather than on organizational, strategic, and stakeholder implications. A recent systematic review identified only 35 relevant studies out of 404 screened publications, revealing substantial gaps across operations management, strategic management, organizational theory, and stakeholder theory [15]. This underscores the need for empirical, multistakeholder research that examines how digital transformation is prioritized, operationalized, and constrained within real-world pharmaceutical development settings. However, existing literature predominantly addresses technology capabilities, regulatory positioning, or FAIR (Findable, Accessible, Interoperable, and Reusable) data governance principles in isolation, rather than integrating stakeholder-derived priorities with implementation barriers across the full transformation journey.

Industry reports characterize macro-level success factors and adoption challenges [5,8,9], while domain-specific studies examine decentralized models, data governance frameworks, or AI application classes [12,16-22]. This fragmented approach limits understanding of how priorities evolve, where tensions emerge, and what collaborative mechanisms can bridge consensus-to-scale gaps. Despite technological maturation and policy evolution, 3 persistent constraints limit transformation success.

Fragmented pilots keep organizations from reaching true digital scale, causing progress to plateau [5,8]. The discourse emphasizes AI acceleration, automation, and generative patterns [12,18-29], while governance, shared measurement, talent development, and cross-functional coordination receive less systematic attention. Evidence remains fragmented as capability layers are examined in isolation [12,16-22] rather than tracing priority evolution or understanding how structural tensions emerge between innovation speed and compliance assurance or between data democratization and privacy protection [30].

A critical knowledge gap exists in the collective understanding of digital transformation priorities. The industry lacks multisource, sequential, mixed methods evidence [31] that maps stakeholder convergence and divergence across outcomes, pathways, and metrics. The industry also needs research that tracks how priorities persist or shift across different engagement modes, from workshops to surveys to expert interrogation.

Finally, the industry needs a better classification of the structural tensions that constrain the movement from consensus to scalable implementation. This gap is particularly significant given the pharmaceutical ecosystem’s stakeholder complexity, encompassing large pharmaceutical companies, midsized organizations, consulting firms, and regulatory bodies, each operating under different constraints and incentive structures [32,33]. To move beyond the predominantly conceptual and technology-centric discussions in the existing literature, this study provides empirical insight into how digital transformation priorities are understood and operationalized across the pharmaceutical value chain.

Unlike prior single-company or technology-focused studies, this work offers a cross-industry, multistakeholder, mixed methods analysis that integrates priorities and implementation barriers across the pharmaceutical value chain. The objectives of this study are to identify and prioritize key digital transformation outcomes, enabling pathways, and measurement domains and to examine the implementation barriers that limit progress. Through a sequential mixed methods design engaging diverse industry stakeholders, this work establishes an evidence-based foundation for understanding where alignment exists, tensions persist, and what factors most influence the ability to scale digital transformation in practice.

## Methods

### Study Design

This study used a sequential mixed methods design to explore end-to-end digital transformation priorities and implementation challenges in the pharmaceutical industry across three themes [32-34]: (1) desired outcomes of digital transformation across the medical product development life cycle, (2) pathways that enable or hinder transformation, and (3) metrics and benchmarks for measuring success.

Three researchers and coauthors (IRR-C, AI, and BE) independently reviewed, coded the data, conducted the analysis, and processed the results. Hereafter, these individuals are referred to as the researchers.

The study comprised 3 interconnected phases. Phase 1 involved a multistakeholder workshop 1 in which participants generated and discussed digital transformation priorities in 3 working groups. The resulting material was coded and consolidated into 19 categories, which were refined into 17 survey topics. A targeted FAIR-data literature search was conducted after workshop 1 to triangulate the theme 1 findings. In phase 2, workshop 1 participants completed an online survey ranking the topics within the 3 themes. In phase 3, workshop 2 used expert panel discussions to examine implementation barriers, trade-offs, and potential resolutions associated with the priorities identified in the earlier phases.

Consistent with the mixed methods design, data collection methods in this study differed across phases as follows: phase 1 used structured contemporaneous notes, phase 2 used an online survey, and phase 3 used audio-recorded and transcribed discussions for in-depth analysis.

The analysis followed the study sequence: workshop 1 generated the categories, the survey quantified their relative priority, and workshop 2 provided implementation-focused explanations of barriers and competing demands. Findings from all phases and the FAIR-data literature review were integrated using convergence and gap analyses. AI-assisted analytic procedures and researcher verification are described in the *Acknowledgments* section and Multimedia Appendix 1.

The design and reporting followed the Standards for Reporting Qualitative Research (SRQR) to support transparency, rigor, and reproducibility in describing qualitative data collection, coding, and adjudication processes [35]. The GRAMMS (Good Reporting of a Mixed Methods Study) framework was used to guide the integration of the qualitative and quantitative components across the sequential mixed methods design [36,37]. The detailed codebook and analysis framework supporting this mixed methods approach are presented in Multimedia Appendix 2.

### Workshop 1

#### Setting, Period, and Sampling

Workshop 1 was held at Novo Nordisk headquarters in Copenhagen, Denmark, in March 2023. The workshop opened with a plenary introduction to the study and theme 1, followed by a breakout discussion in which facilitators guided structured brainstorming and professional writers documented participants’ written and verbal contributions. Participants then reconvened for plenary introductions to themes 2 and 3, with a corresponding breakout discussion after each introduction. The same participants remained in their assigned groups across all 3 breakout sessions. The workshop concluded with a plenary review of the notes from all sessions and collection of participant feedback. Its primary objective was to initiate discussion and evaluation of the 3 core themes guiding the study’s examination of digital transformation. The participants were selected through targeted invitations sent via professional networks, industry associations, and direct outreach to pharmaceutical research and development leaders, consultants, and digital health experts. Eligibility required active involvement in clinical research, digital transformation, or regulatory innovation. Workshop 1 recruitment involved issuing 60 invitations, of which 47 participants attended. Random assignment to 3 working groups (8‐9 participants each) ensured diversity of perspectives and balanced group composition.

#### Data Collection

Workshop 1 comprised 3 parallel working groups, all addressing the 3 themes:

Facilitators guided structured brainstorming sessions, capturing all ideas and experiences in digital worksheets [38]. Each group presented their findings in a plenary session, generating 110 distinct transformation topics. Participants then provided feedback on the presentations.

Written workshop notes, session transcripts, and participant feedback were documented and archived for traceability.

After workshop 1, the researchers conducted a targeted literature search in the Web of Science Core Collection to triangulate theme 1 findings with external evidence (Multimedia Appendix 3) [39]. The search covered publications from 2012 to 2023 and used a high-precision topic query comprising “FAIR data,” “machine-readable data,” “discoverable data,” “interoperable data,” and “democratic data” [40]. The query was combined with the predefined Web of Science life-sciences categories to prioritize precision over recall.

#### Data Analysis

In April 2024, researcher-prepared worksheet notes, plenary summaries, and consolidated outputs from workshop 1 were entered into ChatGPT-4.0 (OpenAI) to generate concise summaries of ideas and provisional groupings of both common and unique categories. These outputs were used only as starting points for the manual review and coding described below.

##### Thematic Coding Workflow

The thematic coding process was conducted by the researchers using a staged approach. First, the researchers independently reviewed the workshop 1 materials, including workshop notes, worksheets, and plenary outputs, to identify recurring ideas and emerging patterns directly from participant responses. Second, open coding was undertaken by assigning descriptive labels to relevant segments of text. These preliminary codes were reviewed deductively using concepts from the NASSS (Non-Adoption, Abandonment, Scale-Up, Spread, and Sustainability) framework [41], including value, organizational factors, implementation barriers, adoption, and sustainability. The researchers cross-checked workshop 1’s written participant notes and their coded outputs, resolved discrepancies through adjudication meetings, grouped related codes into axial categories, and reviewed the resulting category structure across the 3 working groups to assess consistency and representativeness. This process generated the initial 110 coded categories, which were subsequently used for thematic consolidation and survey-topic development.

ChatGPT-4.0 was used as a supportive analytical tool within this process. The inputs comprised deidentified workshop 1 materials and researcher-developed structured prompts from which the model generated provisional summaries, candidate groupings, and patterns of similarity across the material. These outputs were not treated as final codes or findings. The researchers performed human verification by independently comparing the AI-generated outputs against the original written notes and their coding, modifying, merging, or rejecting AI-generated suggestions before reaching consensus on the final coding framework. Representative inputs and prompts, along with corresponding AI-generated outputs, are provided in Multimedia Appendix 1.

##### Consolidation of Categories Into Themes

The researchers consolidated the workshop 1 categories through iterative comparison and semantic grouping, merging redundant items based on conceptual similarity and stakeholder emphasis. This process yielded both common and unique categories, which were subsequently refined into survey topics.

ChatGPT-4.0 supported this consolidation by using structured researcher-developed prompts and researcher-derived categories as inputs to generate provisional semantic groupings. These outputs were reviewed and refined by the researchers following the verification, adjudication, and consensus process described in the thematic coding workflow above. Representative prompts, inputs, and outputs are provided in Multimedia Appendix 1.

##### FAIR Data Literature Analysis

Search results were reviewed descriptively to characterize publication frequency and recurring institutional contributors in the pharmaceutical and biomedical research landscape. Researchers counted publications by year and grouped affiliation metadata using standard Web of Science fields. This procedure was used only to contextualize the workshop 1 findings.

### Survey

#### Setting, Period, and Sampling

The follow-up survey was administered online between March 2023 and April 2023, 4 weeks after workshop 1. It was sent to all 47 workshop 1 participants to preserve a shared frame of reference for prioritizing the categories generated during that workshop and to permit direct within-cohort comparison. The survey was not distributed to new stakeholders; additional stakeholder perspectives were incorporated during workshop 2. Weekly reminder emails were sent to nonrespondents, and the survey closed after the planned 2-week collection window.

#### Data Collection

The online survey instrument was developed from workshop 1 outputs and structured into sections covering participant information, theme prioritization, collaboration preferences, and additional comments. Respondents ranked 17 survey topics (8 in theme 1, 4 in theme 2, and 5 in theme 3) using a ranking scale and provided their name, company affiliation, and role (Multimedia Appendix 4).

Survey responses were collected through an online platform, reviewed for completeness, and exported to Microsoft Excel for analysis. Quality-control procedures included logical consistency checks, verification of participant identities against the invited sample, and data-integrity validation.

#### Data Analysis

Participants ranked the stakeholder-derived topics independently within each of the 3 themes by assigning a unique rank to each topic according to its relative priority within that theme.

The rankings assigned by all participants were tabulated by theme and aggregated for each survey topic to generate an overall ranking score. The aggregated ranking scores were then ordered from highest to lowest within each theme to produce the final prioritized ranking of survey topics. Descriptive summaries of the ranking distributions and overall ranking scores were generated to characterize patterns of stakeholder prioritization across respondents. No inferential statistical analyses were performed because the survey was designed to prioritize stakeholder-derived topics rather than to test hypotheses.

The survey rankings were subsequently incorporated into the cross-phase, mixed methods integration as the quantitative component for comparison with the qualitative findings from workshop 1 and with the implementation tensions identified during workshop 2.

### Workshop 2

#### Setting, Period, and Sampling

Workshop 2 was held at the DPHARM (Disruptive Innovations to Modernize Clinical Research) conference in Boston, United States, in October 2024. The workshop opened with an introductory plenary session, followed by panel discussions on each of the 3 themes, with 5 digital-transformation experts participating in each panel. Each panel discussion was followed by an interactive breakout discussion involving the panelists and all participants. A closing plenary presentation summarized the findings across the 3 theme discussions. All workshop content was audio-recorded. The objective was to build on the initial discussions from workshop 1 and the results of the survey through a more refined examination of the study’s core themes and to support the comprehensive implementation-focused analysis described below. Invitations were sent to workshop 1 and survey participants and additional subject-matter experts (conference participants) to preserve continuity with earlier phases while broadening the discussion with implementation, regulatory, and digital health expertise relevant to implementation barriers. A total of 35 participants attended, including 26 from prior phases.

#### Data Collection

Discussions were audio-recorded and automatically transcribed using Zoom’s audio transcription functionality. The researchers reviewed the full transcripts for accuracy before thematic coding. Each panel corresponded to 1 of the 3 themes so that implementation barriers could be examined using the same outcome, pathway, and measurement structures that were used in workshop 1 and the survey [42].

#### Data Analysis

Workshop 2 data were analyzed across themes using the analytic procedures described in the *Integrated Data Analysis* subsection. These procedures included identification and organization of statements relating to implementation barriers, trade-offs, and competing demands; classification and consolidation of related findings; assessment of their prominence; integration with workshop 1 and survey findings through tension, mixed methods, gap, convergence, and evolution analyses.

### Integrated Data Analysis

#### Implementation Tensions Analysis

The researchers derived implementation tensions [41] from the workshop 2 transcripts and survey outputs to identify and characterize pressures, frictions, trade-offs, and competing forces affecting the translation of evidence-based digital transformation into routine operational use. A 5-step analytic procedure was followed:

1. Identification: extraction of statements reflecting conflicting priorities, trade-offs, or competing demands
2. Classification: categorization of retained statements into predefined tension types (value, resource, timeline, stakeholder, and implementation)
3. Consolidation: grouping of related coded statements within each tension type to identify recurrent implementation tensions across discussions
4. Prioritization: an assessment of the relative prominence of tensions based on their frequency and intensity of emphasis within participants’ discussions
5. Cross-phase validation: comparison of the identified tensions with survey priorities to assess convergence and relevance across study phases

This process supported the synthesis and prioritization of dominant implementation tension domains.

ChatGPT-4.0 supported the analysis by using deidentified workshop 2 transcript excerpts, survey outputs, and structured researcher-developed prompts as inputs. AI assistance was used to organize relevant excerpts, provisionally classify statements within the predefined tension types, consolidate related coded statements and recurring patterns, support comparisons of their relative prominence across discussions, and organize comparisons with survey priorities for cross-phase validation. The researchers reviewed all AI-assisted outputs against the original source data and confirmed, modified, or rejected the proposed classifications and groupings through verification and consensus before determining the final tension domains. The model and version, inputs, prompts, and outputs for each AI-assisted step are provided in Multimedia Appendix 1.

#### Mixed Methods Integration and Gap Analysis

The researchers integrated evidence across the 3 study phases to determine whether priorities identified in earlier phases were sustained, refined, or challenged in later phases and to connect stakeholder priorities with implementation realities. Findings were interpreted sequentially and comparatively, rather than as isolated phase-specific results.

Gap analysis was subsequently used to identify discrepancies between consensus priorities and implementation readiness. Workshop 1 aspirations were compared with workshop 2 barrier discussions; missing enablers such as regulatory frameworks and interoperability were mapped; and identified gaps were classified as structural, procedural, or cultural.

ChatGPT-4.0 supported the mixed methods integration using the complete, researcher-prepared analytical outputs from all 3 study phases: workshop 1 qualitative findings, survey results, and workshop 2 qualitative findings, together with structured, researcher-developed prompts. These inputs were provided to the model to generate preliminary cross-phase comparison matrices, convergence summaries, and gap classifications. The AI-generated outputs were reviewed and refined by the researchers through manual comparison with the complete input data to verify accurate representations and calculated values, followed by data adjudication and consensus. Representative prompts, inputs, and outputs are provided in Multimedia Appendix 1

#### Convergence and Evolutionary Analysis

The researchers used convergence analysis to compare findings across workshop 1, the follow-up survey, and workshop 2. Workshop 1 categories were mapped to the 17 survey topics, survey rankings were normalized within each theme, and workshop 2 transcripts were coded for thematic emphasis, contextual relevance, and semantic alignment with the earlier phases.

The researchers reviewed areas of agreement and discrepancy through structured adjudication meetings, with decisions documented for auditability. Integrated comparison matrices were used to classify findings as high convergence, partial convergence, or divergence. This process identified which priorities remained stable across phases, which evolved, and which revealed implementation tensions.

The ChatGPT-4.0 tool outcomes from mixed methods integration and gap analysis were used in this section. Representative prompts, inputs, and outputs are provided in the mixed methods section of Multimedia Appendix 1.

### Ethical Considerations

Based on the researchers’ determination, this study did not constitute human subjects research and did not require institutional review board oversight or formal ethical review under the International Council for Harmonisation (ICH E6 [R3] Good Clinical Practice) guidelines [43] and the US Department of Health and Human Services (HHS) regulations [44] regarding expert key informants; the data collection focused on the participants’ professional knowledge and expert opinions regarding 3 specific themes of this study within the digital transformation field. No private, sensitive, clinical, or personal behavioral health information was solicited or obtained. Because the research generated data about industry-wide trends and concepts rather than data about the individuals themselves, the participants did not serve as “human subjects” under federal definitions. Administrative information (names, company affiliations, and country names) was collected for sample characterization and roster management. To protect participant confidentiality, all individual names and emails were unlinked and removed from the dataset prior to data analysis following the Safe Harbor deidentification criteria [45]. Broad, nonidentifying attributes, including company affiliations and country names, were retained for analysis and reporting. Findings are presented strictly in the aggregate, ensuring that no combinations of characteristics can reasonably identify individual participants. All procedures conformed to institutional and federal benchmarks for nonhuman participants data governance.

## Results

### Participant Characteristics

#### Workshop 1 Participants

For workshop 1, 60 invitations were issued and 47 participants attended. Participants represented large pharmaceutical companies (n=32, 68.1%), midsized pharmaceutical companies (n=3, 6.4%), consulting firms (n=5, 10.6%), and technology or other organizations (n=7, 14.9%; Table S8 in Multimedia Appendix 5).

#### Survey Respondents

The follow-up survey was completed by 26 of 47 (55.3%) participants, with no missing participant identification or company affiliation data. Respondents were from Denmark (n=9), the United States (n=8), the United Kingdom (n=3), Switzerland (n=2), Belgium (n=2), Germany (n=1), and other regions (n=1). By organization type, respondents represented large pharmaceutical companies (n=17, 65.4%), midsized pharmaceutical companies (n=4, 15.4%), small-sized pharmaceutical companies (n=1, 3.8%), and other (academic/standards; n=2, 7.7%; Table S9 in Multimedia Appendix 5).

#### Workshop 2 Participants

Workshop 2 included 35 participants, of whom 26 had participated in prior phases. Participants represented large pharmaceutical companies (18/35, 51.4%), midsized pharmaceutical companies (4/35, 11.4%), clinical research organizations or consulting organizations (3/35, 8.6%), technology or digital health organizations (7/35, 20%), and patient advocacy or other organizations (3/35, 8.6%; Table S10 in Multimedia Appendix 5).

#### Participant Distribution and Continuity Across Phases

Participants represented 8 geographic groupings from Europe and North America and multiple pharmaceutical, academic, consulting, regulatory, and technology organizations. Workshop 1 included 47 participants from 15 companies (large to midsized), 26 (55.3%) participants completed the follow-up survey, and workshop 2 included 35 participants from 25 companies. Because participants could contribute to more than one phase, attendance totals are phase-specific and are not additive. Detailed geographic distribution and participant continuity across phases are provided in Tables S6 and S7 in Multimedia Appendix 5; detailed company representation and phase-specific engagement are provided in Tables S8 to S10 in Multimedia Appendix 5.

### Workshop 1 and FAIR Data

Workshop 1 generated 110 coded categories across 3 working groups. These were consolidated into 19 categories and subsequently refined into 17 survey topics. Tables 1-3 present the detailed categories by theme.

**Table 1.** Workshop 1, theme 1: common and unique categories that impact digitalization.

Type	Category	Content
Common	Access and equity	Better access to medications; diversity and underserved communities; closing the health equity gap; access to data beyond clinical data; access for vulnerable populations
Common	Treatment and outcomes	Curative vs chronic treatment; reduced side effects; personalized outcomes; prevention; treatment outcomes are effective, simple, and high quality; maximize best outcomes
Common	Collaboration and innovation	Seamless access to data; efficient end-to-end drug discovery; in silico trials; collaboration across stakeholders; democratic data; FAIR^a^ data; regulatory standards; regulatory reform; precompetitive collaboration; developing biomarkers; leading vs reacting; higher innovation levels; novel modalities; trials and approvals based on data only; build startups with exceptional focus on data, automation, and decisions; integrated treatments; cross-industry collaboration
Common	Financial considerations	Affordability; reimbursement; reduce financial burden for treatments
Common	Data and automation	Extensive use of data; trusted medicine; beyond the drug-data is high quality; transparency; improve placebo process
Common	Scale and efficiency	Sustainable scale; faster decisions, better therapies; operating models; unburdened health at scale
Unique	Patient-provider relationship	The treatment finds me; pay for performance; I know my patients; you know me at every visit; service is personalized

a FAIR: Findable, Accessible, Interoperable, and Reusable.

**Table 2.** Workshop 1, theme 2: unique categories identified that impact digital transformation.

Type	Category	Content
Unique	Data-centric approach	Data from research to manufacturing; data is the new currency; cross-industry data marketplace; federated data
Unique	Collaboration and partnership	Share industry benchmarks; responsibility and role of vendors; consider vendors as partners; vendors as specialists and not just suppliers; cross-industry learning (eg, Apple, Google, and Banking)
Unique	Automation and standardization	Lab automation; human factors removed and standards introduced
Unique	Data Services and insights	DaaS^a^ in addition to SaaS^b^; consider IaaS^c^
Unique	Advanced learning and analysis	Federated learning

a DaaS: data as a service.

b SaaS: software as a service.

c IaaS: insights as a service.

**Table 3.** Workshop 1, theme 3: common and unique metric-related categories and benchmarks that define a successful digital transformation.

Type	Category	Content
Common	Data-related categories	Data readiness; poor data governance; lack of data; incompatible data; lack of interoperability; restricted access; lack of common terminology; lack of integration; role of data in decision-making; compliance and data; FAIR^a^ use of all data
Common	Collaboration and alignment	Cross-industry collaboration; connecting the right dots; deliberate effort to collect data on underserved populations; user empowerment; clarity of data ownership; share measures among industry; share effective change management strategies; share positive and negative learning; enablers and capabilities; benchmarks
Common	Strategy and value generation	Return on investment; value generation; aligned strategy; simulation culture; what does good look like; where does it make sense to apply AI; legislation after innovation; culture change index; transparency in AI; what is considered competitive?
Common	Organizational and cultural categories	Ownership of driving harmonization; legacy tech; mergers and acquisitions distractions; foundations are missing; organizational silos; ivory tower; health care is hard; culture; how to measure cultural journey?
Common	Ethical and compliance considerations	Lack of legal framework; compliance and AI; publish data on the number of animals tested
Unique	Strategy and value generation	Data reuse
Unique	Benchmarking and measurement	What does good look like; catalog of outcomes and leading indicators

a FAIR: Findable, Accessible, Interoperable, and Reusable.

Across the 3 themes, participants identified patient-centered outcomes, access and equity, data and automation, collaboration, standardization, governance, value generation, and organizational readiness as central dimensions of digital transformation. The detailed content and the common or unique status of each category are shown in Tables 1-3.

The FAIR literature scan identified recurring barriers related to data silos, limited incentives for data sharing, and implementation complexity. It also identified recurring solution patterns centered on common data models, automated metadata, and governance approaches that support more consistent data organization and use (Multimedia Appendix 3).

### Survey

Across the survey themes, treatment and outcomes ranked highest among the desired outcomes (5.92, SD 3.41), data-centric approach ranked highest among the pathways (3.67, SD 2.40), and data improvement metrics ranked highest among the measurement domains (4.04, SD 2.24). The complete ranking results are shown in Table 4.

**Table 4.** Survey topic rankings by theme (mean rank scores, within-theme ranks; n=26)^a^.

Theme	Topic	Mean rank score (SD)	Rank (within theme)
Theme 1	Treatment and outcomes	5.92 (3.41)	1
Theme 1	Access and equity	5.54 (2.87)	2
Theme 1	Data and automation	5.38 (2.88)	3
Theme 1	Collaboration and innovation	4.69 (2.26)	4
Theme 1	Democratic data (FAIR^b^)	4.62 (2.44)	5
Theme 1	Scale and efficiency	4.54 (2.62)	6
Theme 1	Patient-provider relationship	3.31 (2.27)	7
Theme 1	Financial considerations	2.00 (2.83)	8
Theme 2	Data-centric approach	3.67 (2.40)	1
Theme 2	Collaboration and partnership	2.21 (1.04)	2
Theme 2	Automation and standardization	2.08 (1.37)	3
Theme 2	Data services and insights	2.04 (1.34)	4
Theme 3	Data improvement metrics	4.04 (2.24)	1
Theme 3	Strategy and value generation metrics	3.88 (2.19)	2
Theme 3	Collaboration and alignment metrics	3.12 (1.33)	3
Theme 3	Organizational and cultural metrics	2.44 (1.41)	4
Theme 3	Ethical and compliance metrics	1.52 (3.09)	5

a Higher mean rank scores indicate higher priority within the theme.

b FAIR: Findable, Accessible, Interoperable, and Reusable.

### Workshop 2

Workshop 2 results emphasized continuous patient insights, local engagement for underserved populations, affordable, fit-for-purpose tools, and broader ecosystem integration. For pathways, workshop 2 highlighted FAIR across the full value chain, variations in data and digital literacy, interoperability, and change management. For measurement, workshop 2 emphasized comparability over rigid standards, efficiencies associated with governance across processes and data management, and the potential for digital end points to reduce sample sizes and costs.

### Integrative Results Across Workshop 1, the Survey, and Workshop 2

#### Cross-Phase Synthesis

Cross-phase synthesis identified recurring priorities involving patient-centered outcomes, access and equity, FAIR and democratic data, collaboration, innovation, efficiency, and governance-related measurement needs. These priorities were accompanied by implementation barriers involving governance, interoperability, change management, safety standards, competition, and privacy protection. The integrated findings are presented in the following sections on implementation tensions; mixed methods integration and gap analysis; and convergence and evolution.

#### Implementation Tensions

The study identified 5 industry-wide tensions across 3 themes. Each tension represented both a pressure to advance digital transformation and a competing constraint that could limit implementation.

The first tension was between innovation speed and safety standards. Participants described pressure to adopt digital technologies quickly, while regulatory and patient-safety requirements constrained the pace of adoption. The proposed resolution was a balanced approach that maintains safety while accelerating adoption where appropriate.

The second tension was between collaboration and competition. Participants not only supported precompetitive collaboration on common challenges but also identified the need for individual company differentiation and protection of intellectual property. The proposed resolution was to establish clear boundaries between collaborative and competitive areas.

The third tension was between technology adoption and change management. New digital tools and platforms were viewed as opportunities for transformation, but their implementation depended on training, workflow integration, and cultural change. The proposed resolution was an integrated approach that addresses technology and organizational change together.

The fourth tension was between patient preferences and operational efficiency. Participants emphasized individual preferences and diverse patient needs while recognizing the pressures of operational efficiency and cost reduction. The proposed resolution was to use patient-centric approaches that maintain operational viability.

The fifth tension was between data democratization and privacy protection. Industry-wide learning and collaboration were balanced against the protection of patient data and competitive information. The proposed resolution was to apply FAIR data principles with appropriate privacy safeguards.

#### Mixed Methods Integration and Gap Analysis

Table 5 presents the survey ranking scores, workshop 2 coverage percentages, and gap scores for each topic.

**Table 5.** Gap analysis.

Theme topic	Short label	Survey score	Expected (%)^a^	Workshop 2 (min)	Workshop 2 coverage (%)^b^	Gap score (%)
1.1	Access and equity	5.54	15.39	5.51	13.9	–1.49
1.2	Curative/personalized/prevention	5.92	16.44	6.97	17.6	+1.16
1.3	Data and automation outcomes	5.38	14.94	12.34	31.1	+16.16
1.4	Sustainable scale	4.54	12.61	1.22	3.1	–9.51
1.5	Collaboration and innovation	4.69	13.03	5.37	13.5	+0.47
1.6	FAIR^c^/democratic data	4.62	12.83	0.80	2.0	–10.83
1.7	Financial outcomes	2.00	5.56	2.50	6.3	+0.74
1.8	Patient-provider relationship	3.31	9.19	4.94	12.5	+3.31
2.1	Data-centric end-to-end	3.67	36.70	5.20	12.3	–24.40
2.2	Vendor partnerships	2.21	22.10	6.20	14.7	–7.40
2.3	Automation and standardization	2.08	20.80	20.57	48.7	+27.90
2.4	Data services and insights	2.04	20.40	10.24	24.3	+3.90
3.1	Governance/interoperability	4.04	26.93	12.39	31.7	+4.77
3.2	Collaboration and benchmarks	3.12	20.80	7.58	19.4	–1.40
3.3	Strategy, ROI^d^, and value	3.88	25.87	14.93	38.2	+12.33
3.4	Organization and culture	2.44	16.27	4.15	10.6	–5.67
3.5	Ethics and compliance	1.52	10.13	0.00	0.0	–10.13

a Survey score normalized within its theme = (topic score ÷ sum of theme scores) × 100.

b Minutes on a topic within its theme divided by total minutes for that theme. Gap score (%) = workshop 2 coverage (%) − expected (%). Expected (%) sums to 100% per theme, workshop 2 coverage (%) sums to approximately 100% per theme (rounding). Gap scores per theme sum to approximately 0. Given the minutes per topic and survey scores, expected (%) and gap score (%) can be recomputed directly.

c FAIR: Findable, Accessible, Interoperable, and Reusable.

d ROI: return on investment.

Theme 1 concentrated more than expected on data-and-automation outcomes (+16.16%), with additional emphasis on the patient-provider relationship (+3.31%) and a modest lift in financial outcomes (+0.74%) and collaboration (+0.47%). In contrast, FAIR and democratic data (−10.83%) and sustainable scale (−9.51%) received less attention than their survey importance would suggest, and access and equity were slightly below expectations (−1.49%), while curative/personalized/preventive sat near alignment (+1.16%). Overall, the conversation tilted toward near-term data-driven outcome narratives, while foundational stewardship and scalable operating models were comparatively underserved.

Theme 2 devoted substantially more airtime to automation and standardization than expected (+27.90%) and also gave extra focus to data services and insights (+3.90%). Meanwhile, the data-centric end-to-end view, the connective tissue across the life cycle, was underaddressed relative to its priority (−24.40%), and vendor partnerships received less emphasis than their expected share (−7.40%). The conversation favored pragmatic enablers and standards, leaving the integrative, end-to-end orchestration agenda with less coverage.

Theme 3 leaned into strategy, return on investment, and value generation beyond expectations (+12.33%), and data readiness and governance were also covered slightly more than expected (+4.77%). Collaboration and benchmarking landed near alignment (−1.40%), while organization and culture trailed expectations (−5.67%), and ethics and compliance were notably underaddressed relative to their perceived importance (−10.13%). The emphasis pattern suggests a pull toward measurable value and readiness, with ethical and compliance considerations not receiving proportionate conversation time.

#### Convergence and Evolutionary Analysis

Cross-phase convergence was assessed by comparing the workshop 1 categories, survey rankings, and workshop 2 discussion themes. The resulting persistent and evolving categories are described below.

##### Persisted Categories

Several key categories persisted across both workshops, demonstrating their fundamental importance to digital transformation. Access and equity evolved from workshop 1’s focus on better access to medicines, diversity, and underserved communities to workshop 2’s focus on improving representation, local engagement, and patient-first outcomes, showing refinement and practical application of core principles.

Patient-centric outcomes evolved from workshop 1’s focus on personalized outcomes and reducing patient burden to workshop 2’s high-resolution, longitudinal patient view and emphasis on outcomes that matter to patients, demonstrating progression from general concepts to specific implementation approaches.

Innovation and cures evolved from workshop 1’s focus on curative vs chronic, novel modalities to workshop 2’s focus on redesigning end points and bending the arc in rare diseases, showing a progression movement from aspirational goals to practical implementation strategies.

##### Evolved Topics

Some topics evolved significantly between workshops, reflecting stakeholder learning and refinement of concepts. Prevention evolved from workshop 1’s explicit prevention item to workshop 2’s prevention-by-early-detection framing, including continuous monitoring and the simulation of inclusion/exclusion criteria. This evolution demonstrates how digital technologies can transform traditional prevention approaches.

Simplicity and quality evolved from workshop 1’s treatment outcomes are effective, simple, and high quality to workshop 2’s fit-for-purpose, affordable tools examples, including the echo stethoscope. This evolution shows how digital tools can make complex medical procedures more accessible and user-friendly.

##### Disappeared Topics

Several topics that were prominent in workshop 1 did not appear in the workshop 2 discussions, suggesting either deprioritization or integration into broader themes. In silico trials were not mentioned in workshop 2, possibly indicating that this specific technological approach was integrated into broader digital transformation strategies. Operating models were absent from the workshop 2 discussions, suggesting that operational considerations may have been subsumed into other priority areas. Access to data beyond clinical data was absorbed into broader real-world evidence discussions and was not explicitly mentioned, indicating integration rather than abandonment.

### Linking Key Cross-Phase Results

Cross-phase analysis showed substantial convergence in stakeholder priorities, alongside increasing specificity about implementation challenges. Patient-centered outcomes and access and equity emerged as priorities in workshop 1, ranked first and second, respectively, in the survey, and remained prominent in workshop 2. FAIR and democratic data and collaboration similarly persisted from workshop 1 through the survey and workshop 2, while later implementation discussions exposed tensions around privacy, competitive boundaries, and ecosystem integration. Detailed cross-phase results, including implementation tensions and resolution needs, are presented in Table S11 in Multimedia Appendix 5.

Innovation and curative therapies, along with efficiency, automation, and scale, were established as priorities in workshop 1 and further prioritized through the survey, but they became more implementation focused in workshop 2. Challenges involving safety, interoperability, fit-for-purpose tools, organizational change, and scalability became more prominent. Governance and measurement readiness also persisted across phases, with workshop 2 extending earlier findings on data readiness, benchmarking, strategy, and value measurement to include comparability, governance efficiency, and safeguards for data use.

Overall, convergence was stronger around what stakeholders considered important than around how those priorities could be implemented. The principal cross-phase finding was a progression from stakeholder-defined priorities in workshop 1, through quantitative prioritization in the survey, to clearer identification in workshop 2 of the organizational, operational, governance, safety, privacy, and data conditions required for implementation at scale.

## Discussion

### Principal Findings

This sequential mixed methods study identified and prioritized patient-centered outcomes, FAIR-aligned data practices, collaboration, access and equity, efficiency, and reduction of patient burden as core digital transformation priorities across the pharmaceutical value chain. The study also identified structural barriers that impede implementation, extending prior work beyond single-company and technology-centric perspectives.

The findings demonstrate substantial convergence across stakeholders despite differences in organization type, geography, and role within the medical product development ecosystem. This convergence suggests that several digital transformation priorities have evolved from organization-specific ambitions into shared industry objectives. At the same time, the emergence of recurring implementation tensions indicates that the primary challenge facing the sector is no longer identifying opportunities for digital transformation but translating those opportunities into scalable and sustainable implementation.

### Interpretation of Findings and Comparison With Existing Literature

#### Convergence With Broader Evidence

The strong cross-phase convergence observed in this study aligns with prior work demonstrating that digital transformation in life sciences increasingly depends on patient-centered design, interoperable data, and cross-sector collaboration rather than technology adoption alone [15,18,46]. Comparable studies have similarly shown that sustainable digital transformation requires governance, standards, workforce capability, and organizational readiness to evolve alongside technical innovation. Recent work examining digital transformation in drug development has highlighted that implementation success is strongly influenced by organizational alignment, governance structures, and change management rather than technological capability alone [41,47].

The emphasis on access, equity, and reduction of patient burden is also consistent with emerging evidence demonstrating that digital technologies improve participation, inclusivity, and trial accessibility only when they are accompanied by intentional design, community engagement, and consideration of health equity [48-50]. These findings suggest that stakeholders increasingly view digital transformation not solely as a mechanism for operational efficiency but also as an opportunity to improve patient experience, representation, and access to innovation.

Collectively, these observations reinforce the growing consensus that digital transformation should be understood as a multidimensional change process spanning technology, governance, culture, workforce capability, and patient engagement.

#### Interpretation of the Findings

An important observation was the evolution of discussions across study phases. Workshop 1 focused primarily on opportunities, desired outcomes, and future possibilities, whereas workshop 2 concentrated on implementation barriers, competing priorities, governance challenges, and organizational readiness. This progression suggests that the pharmaceutical industry may be entering a more mature phase of digital transformation in which the challenge is no longer establishing the need for change but determining how transformation can be implemented successfully and sustained over time.

This interpretation is consistent with the implementation science literature, which has increasingly demonstrated that transformation initiatives often encounter barriers during adoption, integration, scale-up, and sustainability rather than during initial innovation [41]. Similar observations have been reported in digital health implementation research and sociotechnical transformation frameworks, including the NASSS framework, which highlights how organizational complexity, governance arrangements, stakeholder interactions, and contextual factors frequently determine whether innovations are successfully embedded into routine practice [41].

The findings, therefore, suggest that pharmaceutical digital transformation should be viewed primarily as a sociotechnical challenge rather than a purely technological one. Stakeholders consistently emphasized trust, collaboration, incentives, workforce readiness, regulatory alignment, and organizational capability alongside technological innovation. These findings imply that future value realization may depend less on acquiring new technologies and more on developing the organizational conditions necessary to implement them effectively [46].

#### Contributions to the Field

Unlike studies focused on specific digital modalities and application areas such as DCTs, AI, digital end points, digital therapeutics, or real-world evidence generation [12-14,51,52], this study provides a cross-stakeholder, end-to-end view of digital transformation priorities spanning outcomes, pathways, and measurement domains across the pharmaceutical product development life cycle.

A key contribution is the identification of a practical implementation-tension framework comprising 5 recurring tensions: innovation vs safety, collaboration vs competition, technology vs change management, patient preferences vs efficiency, and data democratization vs privacy. While aspects of these tensions have been discussed separately within the digital health and implementation literature [41], they have rarely been synthesized into a unified framework derived from multiple stakeholder groups across the pharmaceutical ecosystem.

The framework advances understanding by demonstrating that barriers to digital transformation are often systemic rather than technical. The findings suggest that progress depends not only on technology development but also on governance structures, incentive alignment, organizational readiness, leadership commitment, workforce capability, and effective change management. In this sense, the study extends existing literature by providing an implementation-focused perspective that complements technology-focused digital transformation research [41].

#### Implications for Industry, Regulators, and Technology Providers

For industry, the findings reinforce the importance of organizing digital transformation around a small number of durable priorities rather than fragmented technology initiatives [46,53]. The convergence on patient-centered outcomes, FAIR-aligned data, and collaboration suggests that these areas may provide strategic anchors for future transformation efforts.

For regulators, the findings echo growing calls for clearer expectations regarding digital evidence generation, interoperability, data quality, and governance to reduce uncertainty and support responsible innovation [54]. Regulatory clarity may play an increasingly important role in enabling adoption while maintaining appropriate standards for safety, quality, and trust [50,54].

For technology providers, the findings highlight the importance of developing interoperable, low-burden solutions that align with FAIR principles, support integration into existing workflows, and address implementation challenges alongside technical functionality [16,49]. Providers that support organizational adoption and change management may be better positioned to contribute to sustainable transformation than those focused solely on technology deployment [55].

### Limitations

This study used convenience sampling, which may overrepresent organizations already engaged in digital transformation activities. Although the survey achieved a meaningful response rate (26/47, 55.3%), findings may not generalize to all geographic regions, company types, or health care systems. By design, the survey sample was limited to workshop 1 participants to preserve within-cohort continuity for prioritization; broader perspectives were incorporated in workshop 2 through additional subject matter experts. Large pharmaceutical companies were more heavily represented than smaller organizations, potentially influencing the priorities identified.

Qualitative findings were derived from facilitated workshops and may therefore reflect facilitation dynamics, participant engagement levels, and interpretive decisions during synthesis. Survey rankings represent individual participant perspectives rather than formal organizational positions. These limitations are common within exploratory mixed methods research [32,34] and were partially mitigated through triangulation across workshop 1, the survey, and workshop 2.

### Conclusions

Digital transformation in pharmaceuticals is constrained less by disagreement about priorities than by the difficulty of translating those priorities into scalable implementation. Progress will require harmonized standards, regulatory clarity, workforce capability, effective governance, and sustained collaboration among industry, regulators, standards organizations, technology providers, health care systems, and patient communities.

Future research should evaluate which governance, collaboration, and implementation models are most effective for resolving the recurring tensions identified in this study and enabling sustainable transformation across the life sciences ecosystem.

## Supplementary material

10.2196/91531 Multimedia Appendix 1 Use of generative AI assisted analysis.

10.2196/91531 Multimedia Appendix 2 Detailed codebook and analysis framework.

10.2196/91531 Multimedia Appendix 3 Additional data analysis.

10.2196/91531 Multimedia Appendix 4 Survey instrument and methodology.

10.2196/91531 Multimedia Appendix 5 Participant characteristics and cross-phase findings.

## Abbreviations

DCT: decentralized clinical trial
DPHARM: Disruptive Innovations to Modernize Clinical Research
FAIR: Findable, Accessible, Interoperable, and Reusable
GRAMMS: Good Reporting of a Mixed Methods Study
HHS: Health and Human Services
ICH: International Council for Harmonisation
NASSS: Non‑Adoption, Abandonment, Scale‑Up, Spread, and Sustainability
SRQR: Standards for Reporting Qualitative Research
