# Supplementary material for: Priorities and Implementation Barriers in End-to-End Digital Transformation in the Pharmaceutical Industry: Sequential Mixed Methods Analysis
Source: J Med Internet Res. 2026 Oct 9;28:e91531. doi: 10.2196/91531 (PMC13651978; doi:10.2196/91531)
Supplement: Supplementary file 1 [file jmir-v28-e91531-s001.docx]

## Appendix 5. Use of Generative AI Assisted Analysis

This study used the large language model ChatGPT-4.0 (OpenAI) as a generative AI tool to assist three researchers with data analysis, preliminary clustering, and convergence mapping based on de-identified workshop notes, survey outputs, and literature search materials; all model outputs were treated as provisional and were independently checked, corrected, verified, validated, and finalized.

### Workshop 1 Thematic Coding Workflow and Consolidation of Categories into Themes

#### Example Input (RAW data)

Excerpt Theme 1 ("Desired outcomes for digitalization"):

- Better access to meds
- Curative vs. chronic treatment
- Diversity and underserved communities
- Seamless access to data
- Efficient end to end drug discovery
- In Silico Trials
- Collaboration across stakeholders
- Democratic data
- FAIR data
- Access to data beyond the clinical data
- Closing the health equity gap
- Cure is a goal
- Sustainable scale
- Faster decisions better therapies
- Regulatory standards
- Regulatory reform
- Pre-competitive collaboration
- Operating models
- Developing biomarkers
- Leading vs reacting
- Affordability
- Reimbursement
- Reduce financial burden for treatments
- The treatment finds me
- Personalized outcomes
- Pay for performance
- I know my patients
- You know me at every visit
- Service is personalized
- Regulatory collaboration
- Higher innovation levels
- Novel modalities
- Reduce financial burden for treatments
- Build startup with exceptional focus on data, automation, and decisions
  Improve placebo process
- Trials and approvals based on data only
- Value for patients and society
- Trusted medicine
- Unburdened health at scale
- Focus on cure
- Integrated Treatments
- Transparency
- Access for vulnerable population

#### Example Prompt

You are assisting with qualitative analysis of Workshop 1 on "Desired outcomes for digitalization across the lifecycle of the medical product development value chain that we should aspire to."
I will paste de-identified bullet points from all three working groups for Theme 1. Each line is a participant contribution.
**Tasks**

1. Generate a concise high-level summary (1 short paragraph) of the main outcome areas participants are aiming for.
2. Group the bullets into **provisional categories** that could later be refined into survey topics. For each category:
   - Provide a short label (2-4 words).
   - List 3-6 representative bullets from the input.
   - Mark whether you think this category is **Common** (likely to appear across groups) or **Unique**.
3. Do **not** invent new ideas; stay close to the wording in the bullets.

Output in:

- "Provisional categories" section with a table: [ID] [Label] [Common/Unique] [Representative bullets].

Here are the Theme 1 bullets:
<PASTE BULLETS ABOVE PLUS THE REMAINING THEME 1 ITEMS HERE>

#### Example Output

**Provisional categories**

| **ID** | **Label** | **Common/Unique** | **Representative bullets** |
| --- | --- | --- | --- |
| C1 | Access & Equity | Common | Better access to meds; Diversity and underserved communities; Access for vulnerable population; Closing the health equity gap; Access to data beyond the clincal data |
| C2 | Treatment & Outcomes | Common | Curative vs. chronic treatment; Cure is a goal; Treatment outcomes are effective, simple, high quality; Reduced healthcare burden; Novel modalities |
| C3 | Data & Automation | Common | Seamless access to data; Efficient end to end drug discovery; Beyond the drug - data is high quality; Trusted medicine; Improve placebo process |
| C4 | FAIR/Democratic Data | Common | Democratic data; FAIR data; Access to data beyond the clincal data; Trials and approvals based on data only |
| C5 | Scale & Efficiency | Common | Sustainable scale; Faster decisions better therapies; Unburdened health at scale; Operating models |
| C6 | Collaboration & Innovation | Common | Collaboration across stakeholders; Pre-competitive collaboration; Regulatory collaboration; Higher innovation levels; Leading vs reacting |
| C7 | Financial Considerations | Unique | Affordability; Reimbursement; Reduce financial burden for treatments; Pay for performance |
| C8 | Patient-Provider Relationship | Unique | The treatment finds me; I know my patients; You know me at every visit; Service is personalized |

These provisional categories were then refined by human coders into the consolidated Theme 1 topics shown in Table 3 and used to construct the survey instrument.

### FAIR Data Literature Scan (Theme 1, secondary analysis)

#### Example Input (search strategy)

Excerpt from "FAIR Methodology".
TS=("FAIR data" OR "machine-readable data" OR "discoverable data" OR "interoperable data" OR "democratic Data")
AND (Health Care Sciences Services or Neurosciences or Biochemistry Molecular Biology … or Obstetrics Gynecology or Orthopedics or Pathology or Physics Fluids Plasmas)
The results of the search, around FAIR data (and a few synonyms)… identified several publications. The top 5 abstracts from the cumulated data and review papers were reviewed based on ChatGPT-4.0.
Assume the analyst pasted the 5 abstracts below this block into the chat.

#### Example Prompt

You are supporting a targeted literature scan on FAIR data in life sciences, used to triangulate Workshop 1 Theme 1 "Democratic data through FAIR".
I will provide:

1. The Web of Science topic search used (see above).
2. Five de-identified abstracts from life-science articles about FAIR data and related concepts.

**Tasks**

1. For each abstract, write a 2-3 sentence summary covering: (a) main objective, (b) key data-related barriers, (c) key solution patterns.
2. Produce an integrated synthesis that lists:
   - Recurring barriers to FAIR-aligned data practices in pharma/biomedicine.
   - Recurring solutions/enablers.
   - Which types of organizations (e.g., large pharma, consortia, academia) are most visible in the abstracts.
3. Map each barrier/solution back to one or more Workshop 1 outcome areas: Access & Equity, Treatment & Outcomes, Data & Automation, FAIR/Democratic Data.

Output:

- Part A: Table per abstract [ID] [Objective] [Key barriers] [Key solutions] [Lead stakeholder type].
- Part B: Bullet-point synthesis under Barriers, Solutions, Leading contributors, Link to stakeholder outcomes.

Do not invent numerical results; stay at the level of qualitative patterns.
Here are the five abstracts:
<PASTE TOP 5 FAIR ABSTRACTS HERE>

#### Example Output

**Part A - per-abstract summaries (excerpt)**

| **ID** | **Objective** | **Key barriers** | **Key solutions** | **Lead stakeholder type** |
| --- | --- | --- | --- | --- |
| A1 | Describe a FAIR implementation roadmap for pharma R&D datasets. | Heterogeneous legacy systems, fragmented metadata. | Common data models, harmonized ontologies, governance boards. | Large pharmaceutical company |
| A2 | Evaluate FAIR data practices in academic biobanks. | Limited incentives, unclear data-sharing policies. | Centralized governance, cost-sharing consortia, clear licensing. | Academic consortia |
| A3 | Propose architecture for interoperable clinical research data. | Incompatible EHR formats, manual curation burden. | Automated metadata capture, API-based interoperability hubs. | Hospital network |
| A4 | Assess impact of FAIR principles on translational research. | Siloed pre-clinical and clinical repositories. | Cross-domain identifiers, integrated catalogues. | Mixed public-private |
| A5 | Discuss ethical aspects of democratizing life-science data. | Privacy concerns, unequal access to infrastructure. | Tiered access models, transparent governance. | Policy/ethics scholars |

**Part B - integrated synthesis (excerpt)**

- **Recurring barriers**
  - Fragmented legacy systems and heterogeneous formats that prevent seamless data reuse across pre-clinical, clinical, and real-world settings.
  - Weak incentives and resourcing for long-term FAIR infrastructure, especially in academic and biobank contexts.
  - Metadata gaps and lack of common identifiers that limit discoverability and interoperability.
- **Recurring solutions/enablers**
  - Adoption of shared ontologies and common data models, often coordinated through cross-organizational governance structures.
  - Automated or semi-automated metadata generation and data catalogues to improve findability and reuse.
  - Multi-stakeholder consortia that distribute costs and establish clear, transparent policies for data access and reuse.
- **Link to Workshop 1 outcomes**
  - Barriers around silos and metadata directly affect **Data & Automation** and the ability to achieve **sustainable scale**.
  - Governance and consortium-based solutions underpin the Workshop 1 category **Democratic data through FAIR**, and indirectly support **Access & Equity** by enabling broader data sharing for underserved populations.

### Implementation Tensions (Workshop 2 panels + survey)

#### Example Input

1. **Workshop 2 transcript excerpt** (Theme 1 panel).
   - "We can deploy remote monitoring quickly, but safety and data quality checks always slow us down."
   - "Patients expect personalized, app-based engagement, while our finance team focuses on cost containment."
   - "Federated data sounds great, but our IT teams cannot yet operationalize it at scale."
2. **Survey Theme 1 and 3 priorities**.

| **Theme 1 Topic (abridged)** | **AvgScore** |
| --- | --- |
| Providing better medicines outcomes that are curative vs. chronic | 5.92 |
| Establishing or improving access and equity outcomes | 5.54 |
| Developing data and automation outcomes with extensive use of AI to help clinical decision making | 5.38 |
| Advocate democratic data through FAIR outcomes (Findable, Accessible, Interoperable, Reusable) | 4.62 |

| **Theme 3 Topic (abridged)** | **AvgScore** |
| --- | --- |
| Measuring data improvement metrics (readiness, governance, interoperability, FAIR use of all data) | 4.04 |

#### Example Prompt

You are helping analyze implementation tensions from Workshop 2 and relate them to survey priorities.
**Inputs**

- The transcript from Workshop 2 panels.
- Survey mean scores for the related topics from Themes.

**Tension types** (use exactly one primary type per statement):

1. Value tension - competing objectives (e.g., innovation speed vs. safety).
2. Resource tension - limited budget, staff, time.
3. Timeline tension - speed vs. quality.
4. Stakeholder tension - different stakeholder priorities.
5. Implementation tension - concept vs. operational reality.

**Tasks**

1. Turn each Workshop 2 conversation into a row in a table with columns:
   [ID] [Statement] [Primary tension type] [Short label] [Intensity (Low/Medium/High)] [Related survey topic(s)] [Survey priority (High/Medium/Low within theme)].
2. Base intensity on how strong the conflict appears in the phrasing (here you only have the statements, so use your best qualitative judgement).
3. Use the survey mean scores to classify priority as High (top 2 scores in a theme), Medium (middle scores), or Low (bottom scores).

Do not change the wording of the statements beyond light grammatical cleaning.

#### Example Output

**Tensions table**

| **ID** | **Statement** | **Primary tension type** | **Short label** | **Intensity** | **Related survey topic(s)** | **Survey priority** |
| --- | --- | --- | --- | --- | --- | --- |
| T1 | We can deploy remote monitoring quickly, but safety and data quality checks always slow us down. | Value | Innovation speed vs. safety | High | Developing data and automation outcomes with extensive use of AI; Measuring data improvement metrics | High (both) |
| T2 | Patients expect personalized, app-based engagement, while our finance team focuses on cost containment. | Stakeholder | Patient expectations vs. cost control | High | Providing better medicines outcomes; Establishing or improving access and equity outcomes | High |
| T3 | Federated data sounds great, but our IT teams cannot yet operationalize it at scale. | Implementation | Federated data: concept vs. practice | Medium | Advocate democratic data through FAIR outcomes; Measuring data improvement metrics | Medium/High |

### Mixed-methods Integration (Workshop 1 + survey + Workshop 2)

#### Example Input

1. **Link between Workshop 1 categories and survey topics**

| **Topic ID** | **Survey Theme 1 topic (abridged)** | **Linked Workshop 1 categories** |
| --- | --- | --- |
| O1 | Providing better medicines outcomes that are curative vs. chronic | Curative vs. chronic treatment; Cure is a goal; Focus on cure; Treatment outcomes are effective, simple, high quality |
| O2 | Establishing or improving access and equity outcomes | Better access to meds; Diversity and underserved communities; Closing the health equity gap; Access for vulnerable population |
| O3 | Developing data and automation outcomes with extensive use of AI | Seamless access to data; Efficient end to end drug discovery; Extensive use of data; Trusted medicine; Beyond the drug - data is high quality |

2. **Survey scores** (Theme 1).

| **Topic ID** | **AvgScore** |
| --- | --- |
| O1 | 5.92 |
| O2 | 5.54 |
| O3 | 5.38 |

3. **Workshop 2 coded summaries** (paraphrased from transcript).

- For O1: Panelists repeatedly framed digital health technologies in terms of "outcomes that matter to patients," including curative treatments, fewer side effects, and improved quality of life.
- For O2: Discussions emphasized burden reduction and inclusion of underserved communities, but noted persistent barriers related to protocol complexity and site capacity.
- For O3: Experts highlighted both enthusiasm for AI-supported automation and significant challenges around data quality, governance, and change management.

#### Example Prompt

You are integrating evidence across three phases of a sequential mixed-methods study.
**Inputs**

- Table linking Theme 1 survey topics (O1-O3) to Workshop 1 qualitative categories.
- Survey mean scores.
- Short coded summaries of how each topic featured in Workshop 2 discussions.

**Tasks**

1. For each Topic ID (O1-O3), create an integrated row with:
   [Topic ID] [Theme] [Integrated description] [Survey priority (High/Medium/Low within theme)] [Workshop 2 implementation signal] [Convergence level (High/Partial/Divergent)].
2. Treat the three highest-scoring Theme 1 topics as **High** priority.
3. Define convergence level as:
   - High = strong emphasis and similar framing across all phases.
   - Partial = topic appears but with different emphasis or framing.
   - Divergent = clear mismatch between survey priority and implementation discussion.

Use only the information provided; do not introduce new topics.

#### Example Output

Integrated table:

| **Topic ID** | **Theme** | **Integrated description** | **Survey priority** | **Workshop 2 implementation signal** | **Convergence level** |
| --- | --- | --- | --- | --- | --- |
| O1 | Theme 1 - Treatment & Outcomes | Stakeholders consistently prioritized curative, simple, high-quality treatments and reduced side effects as outcomes that matter most to patients. | High | Panelists framed nearly all digital use cases in terms of their ability to improve patient-relevant outcomes. | High |
| O2 | Theme 1 - Access & Equity | Access and equity outcomes, including reaching underserved and vulnerable populations, were ranked very highly and grounded in Workshop 1 categories on diversity and access. | High | Workshop 2 discussions acknowledged access barriers (e.g., protocol complexity, site capacity), but solutions were less concrete. | Partial |
| O3 | Theme 1 - Data & Automation | Participants valued extensive, trusted use of data and automation to enable better decisions and "beyond the drug" approaches. | High | Implementation discussions highlighted enthusiasm for AI and automation but also substantial challenges in data quality, governance, and change management. | Partial |

### Gap Analysis (priority vs Workshop 2 coverage)

#### Example Input

Exported a table comparing normalized survey scores with approximate proportion of Workshop 2 discussion time per topic.

| **Topic ID** | **Theme** | **Short label** | **Survey Score (% of Theme 1 priority)** | **Workshop 2 coverage (% of Theme 1 discussion time)** |
| --- | --- | --- | --- | --- |
| O1 | Treatment & Outcomes | Patient-relevant outcomes | 22% | 24% |
| O2 | Access & Equity | Access and equity | 21% | 15% |
| O3 | Data & Automation | Data and automation outcomes | 20% | 28% |
| O4 | FAIR/Democratic Data | FAIR/democratic data | 17% | 10% |

#### Example Prompt

You are conducting a simple gap analysis comparing survey-based priority weights with the share of Workshop 2 discussion devoted to each Theme 1 topic.
**Input table**: Topic ID, Theme, Short label, Survey Score (%), Workshop 2 coverage (%).
**Tasks**

1. For each topic, calculate Gap = Workshop 2 coverage - Survey Score.
2. Classify each topic as:
   - "Over-emphasized" if Gap ≥ +5 percentage points.
   - "Under-emphasized" if Gap ≤ −5 percentage points.
   - "Roughly aligned" otherwise.
3. Suggest a likely gap type (structural, procedural, cultural) based on the short label and what you know from the manuscript about barriers (e.g., governance vs. change management).
4. Output an updated table with [Topic ID] [Short label] [Survey Score] [Coverage] [Gap] [Gap classification] [Likely gap type].

Do not alter the input percentages.

#### Example Output

Gap table:

| **Topic ID** | **Short label** | **Survey Score (%)** | **Coverage (%)** | **Gap (%)** | **Gap classification** | **Likely gap type** |
| --- | --- | --- | --- | --- | --- | --- |
| O1 | Patient-relevant outcomes | 22 | 24 | +2 | Roughly aligned | Structural/procedural |
| O2 | Access and equity | 21 | 15 | −6 | Under-emphasized | Structural |
| O3 | Data and automation outcomes | 20 | 28 | +8 | Over-emphasized | Structural |
| O4 | FAIR/democratic data | 17 | 10 | −7 | Under-emphasized | Structural |
