# Supplementary material for: Priorities and Implementation Barriers in End-to-End Digital Transformation in the Pharmaceutical Industry: Sequential Mixed Methods Analysis
Source: J Med Internet Res. 2026 Oct 9;28:e91531. doi: 10.2196/91531 (PMC13651978; doi:10.2196/91531)
Supplement: Supplementary file 2 [file jmir-v28-e91531-s002.docx]

## Appendix 1: Detailed Codebook and Analysis Framework

### Thematic Analysis Codebook

#### Open Coding Categories

The primary categories are as follows:

1. **Data & Technology** - Digital tools, platforms, and data management. This category captured all discussions about the technical infrastructure needed for digital transformation, including artificial intelligence systems, data analytics platforms, and the digital tools that pharmaceutical companies use to manage their operations and research processes.
2. **Patient Experience** - Patient outcomes, preferences, and engagement. This category focused on how digital transformation affects patients directly, including their experiences with healthcare, their preferences for different types of digital health technologies, and how well patients engage with new digital solutions.
3. **Access & Equity** - Healthcare access, health disparities, and affordability. This category addressed the fundamental question of who benefits from digital transformation, covering issues like whether digital health solutions are accessible to all patients regardless of their socioeconomic status, geographic location, or cultural background.
4. **Regulatory & Innovation** - Regulatory frameworks, innovation processes, and collaboration. This category examined how government regulations either help or hinder digital innovation in the pharmaceutical industry, and how different organizations work together to develop new approaches to drug development and healthcare delivery.
5. **Operational Efficiency** - Process optimization, decision-making, and resource management. This category looked at how digital transformation helps pharmaceutical companies work more efficiently, make better decisions faster, and manage their resources more effectively throughout the drug development process.

#### Axial Coding Framework

The theme relationships are as follows:

1. **Causal Relationships:** How themes influence each other. We examined how one theme directly causes or influences another. For example, we found that data quality directly affects patient outcomes, and that regulatory changes influence how quickly companies can adopt new technologies.
2. **Temporal Relationships:** Theme evolution over time. We tracked how themes changed and developed throughout our three-phase study, from the initial brainstorming in Workshop 1, through the prioritization in the survey, to the deep-dive discussions in Workshop 2.
3. **Contextual Relationships:** How themes relate to industry context. We analyzed how themes fit within the broader pharmaceutical industry context, understanding that what works for a large multinational company might not work for a smaller, more specialized organization.
4. **Stakeholder Relationships:** How different groups relate to themes. We examined how different types of organizations (large pharma, mid-sized companies, consulting firms, academic institutions) viewed and prioritized the same themes differently based on their unique perspectives and constraints.

#### Selective Coding Criteria

The convergence indicators are as follows:

1. **Frequency:** Number of groups mentioning theme. We counted how many of the three working groups in Workshop 1 mentioned each theme, with themes appearing in all three groups considered most important.
2. **Intensity:** Strength of emphasis or discussion. We measured how passionately participants discussed each theme, with themes that generated heated debate or strong emotional responses considered more significant.
3. **Consistency:** Similarity of theme expression across groups. We looked for themes that were expressed in similar ways across different groups, suggesting they represented fundamental industry concerns rather than specific organizational interests.
4. **Priority:** Explicit prioritization or ranking. We paid special attention to themes that participants explicitly ranked as most important in the follow-up survey, as these represented their considered judgments rather than spontaneous brainstorming.

### Content Analysis Framework

#### Tension Identification Criteria

The tension types are as follows:

1. **Value Conflicts** - Competing priorities or objectives. These tensions arise when organizations must choose between different important goals. For example, the tension between innovation speed (wanting to adopt new technologies quickly) and patient safety (ensuring new technologies don't harm patients) represents a fundamental value conflict.
2. **Resource Conflicts** - Limited resources or competing demands. These tensions occur when organizations have limited time, money, or personnel and must decide which digital transformation initiatives to pursue. For example, should they invest in patient-facing technologies or back-office efficiency improvements?
3. **Timeline Conflicts** - Speed vs. quality trade-offs. These tensions emerge when organizations feel pressure to move quickly but also want to do things right. For example, should they implement a digital solution quickly to gain competitive advantage, or take more time to ensure it's properly integrated and tested?
4. **Stakeholder Conflicts** - Different group interests or perspectives. These tensions arise when different groups within or outside the organization have conflicting interests. For example, patients might want more personalized care while payers want cost-effective solutions.
5. **Implementation Conflicts** - Theory vs. practice challenges. These tensions occur when theoretical solutions don't work in practice. For example, a digital health solution might work perfectly in a controlled environment but fail when implemented in real-world conditions with diverse patient populations.

#### Consensus Mapping Framework

The consensus levels are as follows:

1. **High Consensus:** Universal agreement across all groups. Themes where all participants agreed, such as the need for patient-centric approaches and the importance of data standards. These represented fundamental principles that transcended organizational differences.
2. **Medium Consensus:** Majority agreement with some dissent. Themes where most participants agreed but some had different views, such as the specific approaches to regulatory reform or the best ways to measure digital transformation success.
3. **Low Consensus:** Mixed opinions or significant disagreement. Themes where participants had very different views, such as the optimal balance between collaboration and competition, or the most effective approaches to change management.
4. **No Consensus:** Clear disagreement or conflicting positions. Themes where participants held fundamentally opposing views, such as whether certain types of data sharing should be mandatory or voluntary.

### Quality Assurance Procedures: Member Checking Process

The validation steps are as follows:

1. **Theme Review:** Participants review identified themes. We went back to workshop participants and asked them to review the themes we had identified to make sure we hadn't missed anything important or misunderstood their perspectives.
2. **Interpretation Validation:** Confirm theme interpretations. We asked participants to confirm that our interpretations of what they said were accurate and that we understood their meaning correctly.
3. **Priority Confirmation:** Validate theme prioritization. We asked participants to confirm that our understanding of which themes were most important aligned with their views and that our priority rankings reflected their actual preferences.
4. **Feedback Integration:** Incorporate participant feedback. We incorporated participant feedback into our final analysis, ensuring that our findings truly reflected the collective wisdom of the group rather than our own interpretations.

### Transparency of AI-Supported Analysis

The tools and safeguards are as follows:

1. **AI Tooling:** ChatGPT-4.0 was used to assist with summarization, clustering, and drafting language for figures/tables. We used artificial intelligence to help process the large amount of qualitative data from our workshops, but we were transparent about this use and ensured human oversight throughout the process.
2. **Human Oversight:** All AI-supported outputs were reviewed and validated against sources prior to inclusion. Every piece of content generated with AI assistance was carefully reviewed by human researchers and checked against our original source materials to ensure accuracy and faithfulness to what participants actually said.
3. **Traceability:** Where AI was used to structure content (e.g., headings, concise summaries), numerical values and factual statements were cross-checked with data sources. We maintained clear records of where AI was used and ensured that all factual information, statistics, and numerical data were verified against our source documents before being included in our analysis.
