# Supplementary material for: Priorities and Implementation Barriers in End-to-End Digital Transformation in the Pharmaceutical Industry: Sequential Mixed Methods Analysis
Source: J Med Internet Res. 2026 Oct 9;28:e91531. doi: 10.2196/91531 (PMC13651978; doi:10.2196/91531)
Supplement: Supplementary file 3 [file jmir-v28-e91531-s003.docx]

## Appendix 3: Additional Data Analysis

### Stakeholder Analysis: Evolution Patterns

The theme development patterns were as follows:

1. **Workshop 1 to Survey:** Strong theme continuity (100% response rate). Themes identified in Workshop 1 were strongly validated in the follow-up survey, with 26 out of 47 participants responding.
2. **Survey to Workshop 2:** Focus shifts from "what" to "how" and barriers. While Workshop 1 and the survey focused on identifying priorities, Workshop 2 shifted to exploring implementation challenges and barriers.
3. **Overall:** 35 themes to 7 common themes to 3 focus areas to working groups. The analysis process systematically refined themes from initial brainstorming through focused working group formation.

### FAIR Literature Scan (conducted after Workshop 1)

The source and strategy were as follows:

1. **Database:** Web of Science Core Collection. We used Web of Science Core Collection, a comprehensive database of scientific literature, to search for relevant research on FAIR data principles.
2. **Query focus:** FAIR data and close synonyms (e.g., "FAIR data", "machine-readable data", "discoverable data", "interoperable data", "democratic data"). We searched for FAIR data and related terms to ensure we captured all relevant research on data sharing and interoperability principles.
3. **Category filter:** Restricted to life sciences via Web of Science subject categories (precision prioritized over recall). We focused specifically on life sciences research to ensure relevance to the pharmaceutical industry context.
4. **Rationale:** Emphasize precision and domain relevance to validate Workshop 1 Theme 1 signals. We prioritized precision over recall to ensure that the research we found was directly relevant to the themes identified in Workshop 1.
5. **Screening and Abstraction:** Top abstracts from cumulative data and review papers were screened and summarized with ChatGPT-4.0 assistance, followed by human validation. We screened the top abstracts and review papers, using AI assistance to summarize the content, but ensured human validation of all findings.
6. **Outputs:** The FAIR literature scan showed that publication counts were highest for Novartis (n=5), followed by AstraZeneca (n=4), Roche Holding (n=4), AbbVie (n=3), Merck Company (n=3), and Takeda Pharmaceutical (n=2). Annual publication counts were 3 in 2012, 7 in 2013, 8 in 2014, 10 in 2015, 9 in 2016, 12 in 2017, 11 in 2018, 21 in 2019, 27 in 2020, 45 in 2021, 38 in 2022, and 17 in 2023.
