# Supplementary material for: Priorities and Implementation Barriers in End-to-End Digital Transformation in the Pharmaceutical Industry: Sequential Mixed Methods Analysis
Source: J Med Internet Res. 2026 Oct 9;28:e91531. doi: 10.2196/91531 (PMC13651978; doi:10.2196/91531)
Supplement: Supplementary file 4 [file jmir-v28-e91531-s004.docx]

## Appendix 2: Survey Instrument and Methodology

### Survey Design

#### Survey Structure

The survey sections are as follows:

1. **Participant Information** - Name, company, role. This section collected basic demographic information to understand who was responding and ensure we could track responses back to specific participants and organizations.
2. **Theme Prioritization** - Rank workshop themes by importance. This was the core section where participants ranked the 17 consolidated themes from Workshop 1 on scales of 1-8 (for Theme 1) and 1-5 (for Themes 2 and 3) to identify which themes were most important to them.
3. **Collaboration Preferences** - Preferred collaboration approaches. This section explored how participants preferred to work together, including their preferences for meeting formats, communication methods, and collaboration structures.
4. **Additional Comments** - Open-ended feedback and suggestions. This section provided space for participants to share additional thoughts, concerns, or suggestions that didn't fit into the structured questions.

#### Survey Content

End-to-End Digital Strategy Workshop Outputs Survey Shortlisting Focus Topics.

1. **For Theme 1**: Please rank the topics on a scale that closely aligns with an End-to-End Digital Strategy based on your understanding. Move the highest preference to the top of the table.
   1. Establishing or improving access and equity outcomes such as better access to medicines, diversity and underserved communities, closing the health equity gap and access to data beyond clinical trials.
   2. Providing better medicines outcomes that are curative vs. chronic treatment with reducing side effects, personalized, preventing disease, simple and with high quality.
   3. Developing data and automation outcomes with extensive use of data, beyond the drug approach, improved placebo process and transparency.
   4. Able to achieve sustainable scale outcomes with faster decisions, operating models, unburdened health at scale.
   5. Building strong collaboration & innovation outcomes across siloed stakeholders with seamless access to data, efficient end-to-end drug discovery and in silico trials.
   6. Advocate democratic data through FAIR outcomes (Findable, Accessible, Interoperable, Reusable), suit regulatory standards and forum, allowing pre-competitive collaboration, leading vs reacting, boosting higher innovation levels such as novel modalities, potential for trials and approvals based on data only, allowing for integrated treatments.
   7. Providing financial outcomes such affordability, reimbursement gains, and reduced financial burden for treatments.
   8. Developing and enhancing patient-Provider relationship outcomes with opportunities for 'treatment finds me', pay for performance, I know my patients, you know me at every visit and service is personalized.
2. **For Theme 2**: Please rank the topics on a scale that closely aligns with an End-to-End Digital Strategy based on your understanding. Move the highest preference to the top of the table.
   1. Establishing a data-centric approach that brings in data from Research to Manufacturing, data is the new currency, cross industry data marketplace and federated data.
   2. Building strong collaboration & partnership with vendors as partners and specialists, taking learnings from other industries (e.g., Apple, Google, Banking).
   3. Enabling automation and standardization from the lab onwards to remove human factors and build ways of working using standards.
   4. Enabling the use of data services & insights with data as a service (DaaS) in addition to SaaS as well as insights as a Service (IaaS) with advanced learning and analysis leading to more federated learning.
2. **For Theme 3**: Please rank the topics on a scale that closely aligns with an End-to-End Digital Strategy based on your understanding. Move the highest preference to the top of the table.
   1. Measuring data improvement metrics around data readiness, poor data governance, lack of data, incompatible data, lack of interoperability, restricted access, lack of common terminology, lack of integration, role of data in decision-making, compliance and data and FAIR use of all data.
   2. Measuring collaboration and alignment through the number and quality of cross industry collaborations, connecting the right dots, deliberate effort to collect data on underserved populations, user empowerment, clarity of data ownership, sharing of measures amongst industry, sharing of effective change management strategies, sharing positive and negative learning, sharing enablers and capabilities as well as internal benchmarks.
   3. Measuring strategy and value generation such as return on investment, value generation, data reuse, aligned strategy, simulation culture, what does good look like, cataloging outcomes and leading indicators, where does it make sense to apply AI, legislation impacts after innovations, transparency in AI and what is considered competitive.
   4. Measuring organizational and cultural factors including ownership for driving harmonization across the value chain, decisions on legacy tech, M&A distractions, foundational infrastructure and organizational model missing, organizational silos, ivory towers, healthcare is hard, culture change index and the cultural journey.
   5. Measuring ethical and compliance considerations such as lack of legal framework across the value chain, compliance and AI, and publishing data on the number of animals tested.

#### Survey Administration

The survey administration timeline was as follows:

3. **Distribution:** 2-4 weeks post-Workshop 1. We sent the survey 2-4 weeks after Workshop 1 to give participants time to reflect on the discussions while maintaining engagement momentum and ensuring the workshop content was still fresh in their minds.
4. **Reminders:** Weekly reminder emails. We sent weekly reminder emails to encourage participation while being respectful of participants' time and not being overly aggressive in our follow-up.
5. **Closure:** Survey closed after the planned collection window. We closed the survey after a predetermined period to ensure timely analysis and to respect participants' time by not keeping it open indefinitely.
6. **Data Export:** Excel format for analysis. We exported all survey data in Excel format to facilitate quantitative analysis and integration with our qualitative workshop findings.

#### Response Validation

The quality checks were as follows:

1. **Completion Rate:** 26 of 47 invited participants responded (55.3%); among respondents, key fields were fully completed. We achieved a 55.3% response rate, which is typical for industry surveys and indicates strong stakeholder engagement. All respondents completed the key fields with no missing data.
2. **Data Completeness:** No missing data for key fields. We verified that all essential information was captured, including participant identification, company affiliation, and theme prioritization data.
3. **Response Consistency:** Logical consistency in responses. We checked that participants' responses were internally consistent and made logical sense, such as not ranking the same theme as both highest and lowest priority.
4. **Participant Verification:** Confirmation of participant identity. We verified that survey responses came from actual Workshop 1 participants to ensure data integrity and prevent unauthorized responses.

### Survey Results Analysis

#### Descriptive Statistics

The response metrics were as follows:

1. **Total Responses:** 26 participants (55.3% of Workshop 1 participants). We received responses from 26 out of 47 invited participants, representing a strong response rate for an industry survey and ensuring sufficient data for meaningful analysis.
2. **Response Rate:** 55.3% (26/47 participants). This response rate is consistent with typical industry survey participation patterns and indicates strong stakeholder engagement with the digital transformation priorities identified in Workshop 1.
3. **Completion Time:** Average 15-20 minutes. Participants took an average of 15-20 minutes to complete the survey, indicating that it was appropriately sized and not overly burdensome.
4. **Data Quality:** High (no missing data). The survey achieved high data quality with no missing data for key fields, ensuring that our analysis was based on complete information.

#### Priority Analysis

The prioritization methods were as follows:

1. **Ranking Analysis:** Theme importance rankings. We analyzed the numerical rankings participants assigned to each theme to identify which themes were considered most important across the industry.
2. **Frequency Analysis:** Most commonly prioritized themes. We counted how often each theme appeared in the top priority rankings to identify themes that were consistently important across participants.
3. **Subgroup Analysis:** Differences by company type and geography. We analyzed whether different types of organizations (large pharma, mid-sized companies, consulting firms) or different geographic regions (North America, Europe) prioritized themes differently.
4. **Consensus Analysis:** Agreement levels across participants. We measured the level of agreement among participants about theme priorities to understand where the industry was aligned and where there was disagreement.
