# Supplementary material for: Priorities and Implementation Barriers in End-to-End Digital Transformation in the Pharmaceutical Industry: Sequential Mixed Methods Analysis
Source: J Med Internet Res. 2026 Oct 9;28:e91531. doi: 10.2196/91531 (PMC13651978; doi:10.2196/91531)
Supplement: Supplementary file 5 [file jmir-v28-e91531-s005.docx]

## Appendix 4. Supplementary Tables

### Table 6. Geographic Distribution Across All Phases*

| **Country** | **Workshop 1** | **Survey** | **Workshop 2** | **Total**** | **Notes*** |
| --- | --- | --- | --- | --- | --- |
| Denmark | 25 | 9 | 2 | 36 | Host country, Novo Nordisk headquarters |
| USA | 8 | 8 | 28 | 44 | Major pharma market, diverse representation |
| UK | 3 | 3 | 1 | 7 | Academic and consulting presence |
| Switzerland | 2 | 2 | 2 | 6 | Pharma hub, Novartis/Novo Nordisk presence |
| Belgium | 1 | 2 | 1 | 4 | Janssen headquarters |
| Germany | 1 | 1 | 1 | 3 | Boehringer Ingelheim |
| Netherlands | 1 | 0 | 0 | 1 | Viatris presence |
| Other | 6 | 1 | 0 | 7 | Additional representation |

Legend:
*See Tables 8-10 for detailed company representation.
**Totals reflect phase-specific attendance and are not additive across phases; the same individuals may appear in multiple phases.

### Table 7. Participant Continuity and Engagement.

| **Phase** | **Total Participants** | **Unique Companies** | **Response Rate/Attendance** |
| --- | --- | --- | --- |
| **Workshop 1** | 47 | 15 | 100% (in-person) |
| **Survey** | 26 | 13 | 55.3% (26/47) |
| **Workshop 2** | 35 | 25 | 74.5% (35/47) |

Legend: Counts are per‑phase and may include the same participants across phases. More detailed information about participant demographics, company representation, and engagement levels across all study phases is provided in Tables 8-10.

### Table 8: Workshop 1 Participant Overview (N = 47)

| **Company Type** | **Number** | **Percentage** | **Examples of Representative Companies** |
| --- | --- | --- | --- |
| Large Pharmaceutical Companies | 32 | 68.1% | Novo Nordisk (22), J&J/Janssen (2), Roche, AstraZeneca, BMS, Boehringer Ingelheim, GSK |
| Mid-sized Pharmaceutical Companies | 3 | 6.4% | Lundbeck (2), Viatris |
| Consulting Firms | 5 | 10.6% | Accenture (4), BCG |
| Technology/Other Organizations | 7 | 14.9% | Oracle (2), IEEE-SA CTTMN, Novo Nordisk Digital |
| Total | 47 | 100% |  |

Legend:
Number & Percentage: Participants that attended the Workshop 1.

### Table 9: Survey Participant Company Type Distribution (N = 26)

| **Company Type** | **Number** | **Percentage** | **Examples of Representative Companies** |
| --- | --- | --- | --- |
| Large Pharmaceutical Companies | 17 | 65.4% | Novo Nordisk, Janssen, Sanofi, Bristol Myers Squibb, Genentech (Roche), Novartis, Boehringer Ingelheim, Eli Lilly |
| Mid-sized Pharmaceutical Companies | 4 | 15.4% | H. Lundbeck A/S, Daiichi Sankyo, UCB, Regeneron |
| Consulting Firms | 2 | 7.7% | Accenture, Phorix |
| Small-sized Pharmaceutical Companies | 1 | 3.8% | Viatris |
| Other (Academic/Standards) | 2 | 7.7% | IEEE-SA CTTMN, University of Bristol |
| Total | 26 | 100% |  |

Legend:
Number & Percentage: Participants that completed the survey.

### Table 10: Workshop 2 Participant Overview (N = 35)

| **Company Type** | **Number** | **Percentage** | **Examples of Representative Companies** |
| --- | --- | --- | --- |
| Large Pharmaceutical Companies | 18 | 51.4% | J&J, Genentech Roche, Eli Lilly, Pfizer, Sanofi, Biogen, AbbVie, Boehringer-Ingelheim, Novo Nordisk (2), Janssen, Alexion Astrazeneca |
| Mid-sized Pharmaceutical Companies | 4 | 11.4% | Genmab (2), Regeneron (2) |
| CRO/Consulting Organizations | 3 | 8.6% | IQVIA, Citeline, Alira Health |
| Technology/Digital Health | 7 | 20.0% | Medable (2), BullFrog AI, Empatica, Myra EB Systems, Simcere Zaiming |
| Patient Advocacy/Other | 3 | 8.6% | Patient Advocate, IEEE, Former FDA |
| Total | 35 | 100% |  |

Legend:
Number & Percentage: Participants that attended the Workshop 2.

### Table 11. Main cross-phase priorities, implementation tensions, and resolution needs.

| **Cross-phase priority** | **Evidence across phases** | **Related implementation tension** | **Resolution need identified in Workshop 2** |
| --- | --- | --- | --- |
| Patient-centered outcomes | Workshop 1 identified treatment and outcomes and patient-provider relationship categories; the survey ranked treatment and outcomes highest; Workshop 2 emphasized outcomes that matter to patients and continuous patient insight | Patient preferences vs operational efficiency | Patient-centric approaches maintaining operational viability |
| Access and equity | Workshop 1 identified access and equity as a common category; the survey ranked access and equity second; Workshop 2 emphasized local engagement and underserved populations | Patient preferences vs operational efficiency | Patient-centric approaches maintaining operational viability |
| FAIR and democratic data | Workshop 1 identified FAIR data and democratic data within common categories; the survey retained FAIR/democratic data as a prioritized topic; Workshop 2 emphasized FAIR across the full value chain | Data democratization vs privacy protection | FAIR data principles with appropriate privacy safeguards |
| Collaboration | Workshop 1 identified collaboration across stakeholders, pre-competitive collaboration, and cross-industry collaboration; the survey ranked collaboration-related topics highly across outcomes and pathways; Workshop 2 emphasized broader ecosystem integration | Collaboration vs competition | Clear boundaries for collaborative versus competitive areas |
| Innovation and curative therapies | Workshop 1 identified curative therapies, novel modalities, and innovation-related categories; the survey ranked treatment and outcomes highest; Workshop 2 emphasized digital endpoints and practical implementation barriers | Innovation speed vs safety standards | Balanced approach maintaining safety while accelerating adoption |
| Efficiency, automation, and scale | Workshop 1 identified scale and efficiency, operating models, and data and automation; the survey prioritized data-centric approaches and automation-related topics; Workshop 2 emphasized interoperability, affordable fit-for-purpose tools, and automation | Technology adoption vs change management | Integrated approach to technology and organizational change |
| Governance and measurement readiness | Workshop 1 identified governance, data readiness, benchmarking, and strategy/value generation; the survey ranked data improvement metrics and strategy/value metrics highest; Workshop 2 emphasized comparability, governance efficiencies, and readiness | Technology adoption vs change management; data democratization vs privacy protection | Integrated governance approaches and clearer safeguards for data use |
